# Large cooperativity and microkelvin cooling with a three-dimensional optomechanical cavity

**DOI:** 10.1038/ncomms9491

**Published:** 2015-10-09

**Authors:** Mingyun Yuan, Vibhor Singh, Yaroslav M. Blanter, Gary A. Steele

**Affiliations:** 1 Kavli Institute of Nanoscience, Department of Quantum Nanoscience, Delft University of Technology, PO Box 5046, 2600 GA Delft, The Netherlands

## Abstract

In cavity optomechanics, light is used to control mechanical motion. A central goal of the field is achieving single-photon strong coupling, which would enable the creation of quantum superposition states of motion. Reaching this limit requires significant improvements in optomechanical coupling and cavity coherence. Here we introduce an optomechanical architecture consisting of a silicon nitride membrane coupled to a three-dimensional superconducting microwave cavity. Exploiting their large quality factors, we achieve an optomechanical cooperativity of 146,000 and perform sideband cooling of the kilohertz-frequency membrane motion to 34±5 μK, the lowest mechanical mode temperature reported to date. The achieved cooling is limited only by classical noise of the signal generator, and should extend deep into the ground state with superconducting filters. Our results suggest that this realization of optomechanics has the potential to reach the regimes of ultra-large cooperativity and single-photon strong coupling, opening up a new generation of experiments.

## Abstract (web summary)

Optomechanics is the use of light to control the motion of a mechanical resonator, potentially cooling it to the quantum ground state. Here, the authors cool a millimetre-scale silicon nitride membrane to an effective temperature of 34 microkelvin by coupling it to a three-dimensional microwave cavity.

## Graphical abstract

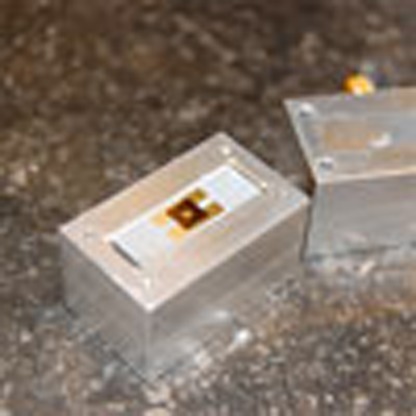

In recent years, cavity optomechanics has been used to realize a wide range of exciting experiments with mechanical resonators, including achieving exquisite measurement precision^1^,^2^,^3^, strong coupling between the mechanical and cavity modes^4^,^5^,^6^, cooling to the quantum ground state^7^,^8^, for microwave amplification^9^,^10^, to entangle propagating microwave photons with mechanical motion^11^, for microwave photon storage^12^,^13^, to observe quantum back-action noise^14^, to generate squeezed light^15^,^16^ and to transduce photons between the optical and microwave domains^17^,^18^,^19^. These successful experiments, performed in the regime of linear optomechanics, were enabled by improvements of the coherence of the optomechanical coupling between light and motion.

In linear optomechanics, the relevant figure of merit for the optomechanical coupling is a parameter called cooperativity. Cooperativity combines the optomechanical coupling rate *g*, the cavity decay rate *κ* and the mechanical decay rate *γ*_m_ into a dimensionless constant 
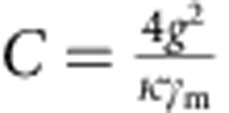
 that quantifies the optomechanical system's efficiency in exchanging photons and phonons^20^. It is similar to the Purcell factor in atomic physics (also often referred to as cooperativity) describing the coupling between cavity fields and atoms. An advantage of linear optomechanics is that compared with the single-photon coupling rate *g*_0_, the multiphoton coupling rate *g* is significantly enhanced, given by 
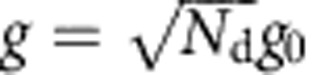
 where *N*_d_ is the number of photons used to drive the cavity. The relevant limit for cooperativity in quantum experiments is the so-called quantum coherent limit, in which the cooperativity is larger than the number of equilibrium thermal quanta in the mechanical resonator. In the quantum coherent limit, an exchange of a photon and phonon occurs faster than the time it takes for a phonon to leak into the mechanical resonator ground state from the thermal bath. Achieving higher cooperativity that is deeper in the quantum coherent limit in linear optomechanics would imply the ability to cool closer to the quantum ground state and the preparation of mechanical quantum states with high fidelity.

Beyond linear optomechanics, the field is striving to reach the limit of single-photon strong coupling, in which interaction of light and mechanical motion is coherent at the level of a single photon. In this limit, the single-photon coupling rate *g*_0_ exceeds both the cavity decay rate *κ* and the mechanical decay rate *γ*_m_ (*g*_0_>*κ*, *γ*_m_). If one could reach this limit, one could take advantage of the intrinsic nonlinearity of the optomechanical coupling at the single-photon level to construct quantum superpositions of mechanical motion using optomechanics with classical light. In current experiments 
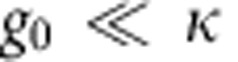
, and existing optomechanical implementations would require significant improvements in *g*_0_ and/or *κ* to approach single-photon strong coupling.

Here we present an optomechanical architecture with large optomechanical coupling that can potentially reach the single-photon strong coupling limit. The design combines two highly coherent elements that are applied for the first time in the microwave optomechanics domain. The first element is a millimetre-sized, nanometre-thick high-stress silicon nitride (SiN_*x*_) membrane, a technology that has demonstrated quality factors up to 1 × 10^7^ at cryogenic temperatures^21^. Such membranes have been used extensively in the optical domain^14^,^16^,^22^,^23^,^24^ and in transducer applications^18^,^19^, but have not yet been explored as an element in a pure microwave optomechanical system. The second optomechanical element is a three-dimensional (3D) microwave cavity, recently popular in the superconducting qubit community for their exceptional coherence times^25^,^26^,^27^. By combining these two elements, we create an optomechanical platform with large cooperativity, demonstrate cooling to the lowest mechanical mode temperature reported to date, and show that this system has the potential to scale to couplings significantly beyond the state of the art.

## Results

### Description and characterization of the device

**Figure 1:**
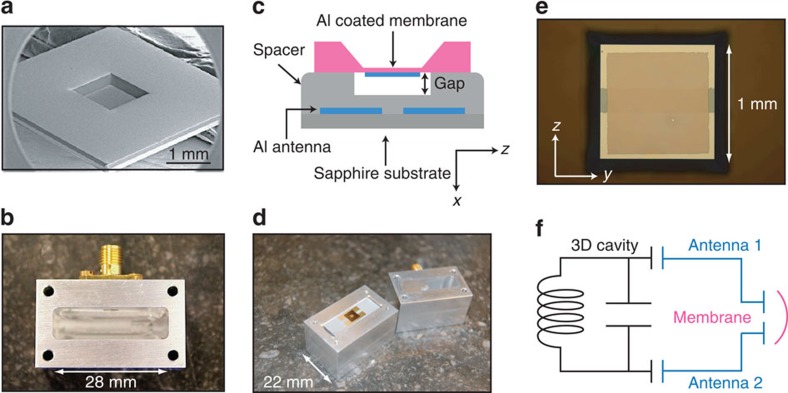
Microwave optomechanics with a 3D superconducting cavity and a millimetre-sized membrane. (**a**) Electron microscope image of a 50-nm-thick SiN_*x*_ membrane. (**b**) One half of the Al cavity with an SMA connector for reflection measurements. The dimensions of the cavity are 28 × 28 × 8 mm. (**c**) Schematic showing the placement of the membrane over the antenna pads. The Al coating of the membrane forms a capacitor with the antennas below. (**d**) A complete assembly. The membrane is positioned in the centre of the cavity, supported by a sapphire substrate patterned with the Al antenna. (**e**) An optical microscope image looking from the top showing the Al-coated membrane and the underlying antenna pads. (**f**) Effective lumped-element model of the cavity and membrane. By changing the effective shunt capacitance, the frequency of the 3D cavity is modulated by the mechanical motion of the membrane.

Figure 1 illustrates the principle of this 3D optomechanical platform. The mechanical resonator is made from high-stress SiN_*x*_ membrane (Fig. 1a) that is metallized with an Al electrode. The cavity itself is an aluminium box (Fig. 1b) in which electromagnetic fields are confined by superconducting walls in all three dimensions. Coupling to the motion of the membrane is achieved by using a flip-chip technique to place the membrane on top of antenna electrodes on a separate substrate (Fig. 1c–e). Figure 1f shows an effective lumped-element circuit model of the assembled cavity and membrane. Optomechanical coupling results from the modulation of the effective shunt capacitance when the membrane displaces, changing the cavity frequency. From finite-element simulations, we estimate a single-photon coupling rate of *g*_0_=(d*ω*_0_/d*x*)·*x*_zpf_=2*π* × 0.36 Hz, where *ω*_0_ is the cavity mode frequency, *x* is the mechanical displacement of the membrane and *x*_zpf_ is the amplitude of its zero-point fluctuation. The simplicity of the assembly of this 3D cavity architecture also makes it attractive for implementing devices such as microwave-to-optical transducers^17^,^18^,^19^ by potentially incorporating an optical fibre into the superconducting box.

**Figure 2:**
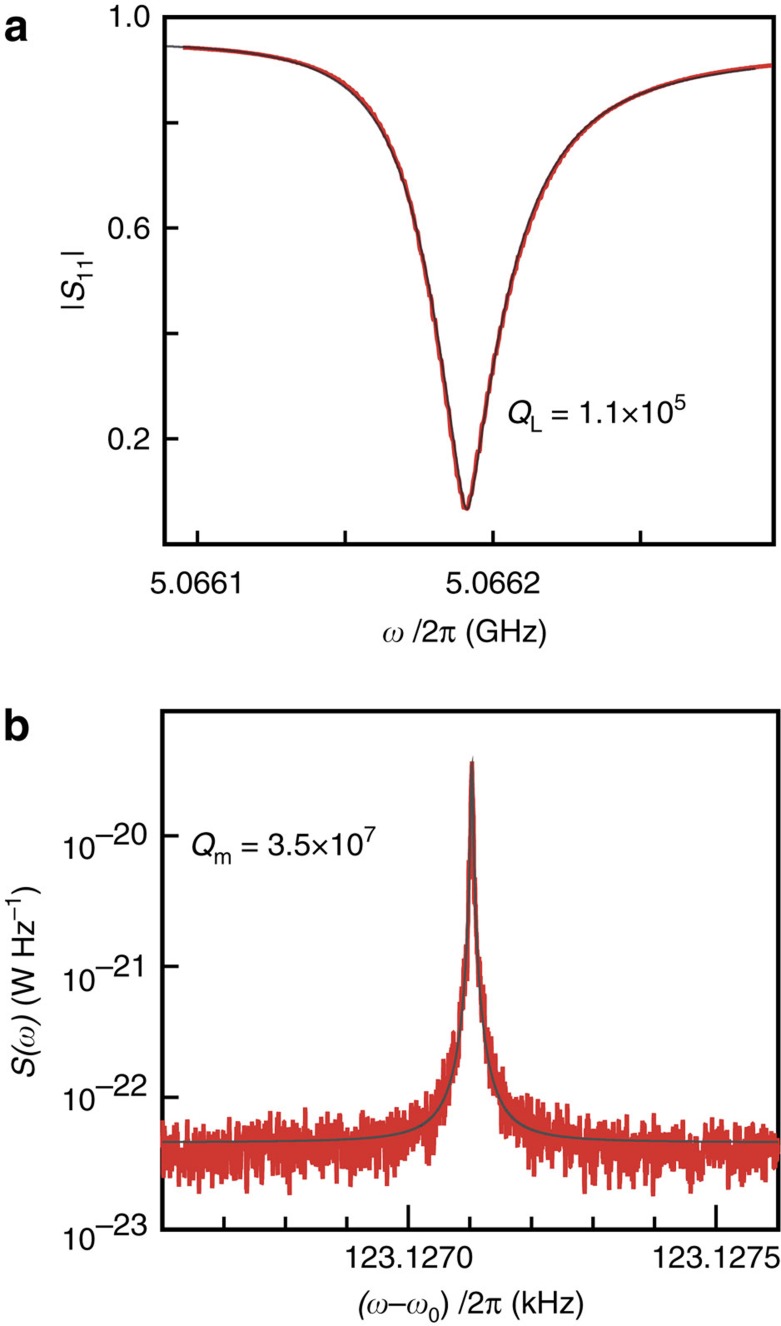
High-quality factors of the microwave cavity and mechanical resonator. (**a**) Reflection coefficient 
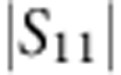
 measurement of the cavity. The loaded quality factor is *Q*_L_=1.1 × 10^5^, corresponding to a total decay rate *κ*=2*π* × 45.5 kHz. Internal dissipation rate is *κ*_0_=2*π* × 23.9 kHz, with internal quality factor *Q*_0_=2 × 10^5^. (**b**) Power spectral density of the thermomechanical motion of the fundamental mode of the membrane *ω*_m_≈2*π* × 123 kHz with linewdith *γ*_m_≈2*π* × 3.5 mHz, corresponding to *Q*_m_=3.5 × 10^7^. Red: data; Grey: fit.

Measurements are performed in a dilution refrigerator with a base temperature of *T*_b_=13 mK (Supplementary Fig. 1 and Supplementary Note 1). Figure 2a shows a measurement of the reflection coefficient 
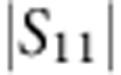
 of the cavity. From a fit to the data, we find a total linewidth of *κ*=2*π* × 45.5 kHz, corresponding to a loaded quality factor of *Q*_L_=1.1 × 10^5^. From power dependence, we find a maximum intracavity photon occupation *N*_max_=1.3 × 10^8^ before the onset of a nonlinear response. The ratio *η*=*κ*_e_/*κ*≈0.48 of the external decay rate *κ*_e_ and *κ* indicates that the cavity is slightly undercoupled (Supplementary Fig. 2 and Supplementary Note 2).

In Fig. 2b, we characterize the mechanical response of the membrane with the cavity using a resonant microwave tone injected at *ω*_0_. The thermomechanical motion of the membrane generates a peak in the sideband power spectral density (PSD) *S*(*ω*) of the microwave field leaving the cavity at a frequency offset Δ*ω*=*ω*_m_ from the carrier signal, shown in Fig. 2b. We find a mechanical resonance frequency of *ω*_m_=2*π* × 123 kHz, consistent with expected fundamental mode frequency of the membrane. A Lorentzian fit yields a linewidth of *γ*_m_=2*π* × 3.5 mHz, corresponding to an ultrahigh mechanical quality factor of *Q*_m_=3.5 × 10^7^, significantly higher than the typical 
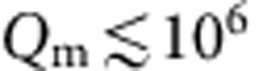
 of membranes used in optomechanical and transducer experiments.

### Large cooperativity

**Figure 3:**
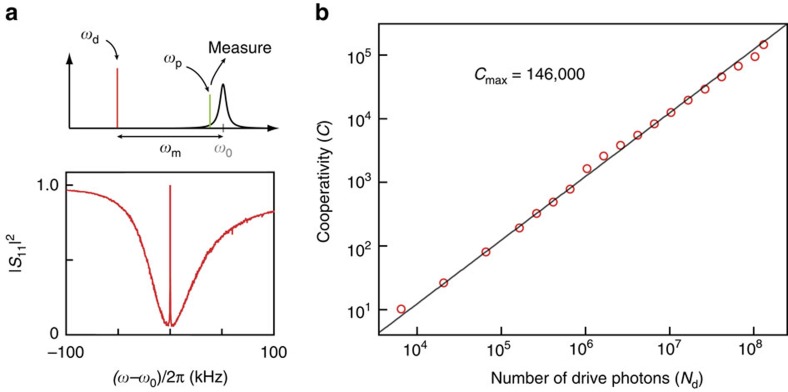
Large cooperativity measured with OMIT. (**a**) Illustration showing the OMIT measurement scheme. The cavity reflection coefficient |*S*_11_| is measured with a weak probe tone (*ω*_p_) while driving the cavity with a second strong tone near the red sideband (*ω*_d_=*ω*_0_−*ω*_m_). A window of transparency appears in the cavity resonance (lower panel) with a width set by the mechanical linewdith. (**b**) Extracted cooperativity *C* versus driving photon number *N*_d_. Grey line: expected linear scaling 
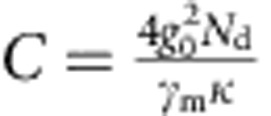
. At maximum *N*_d_, the cooperativity reaches *C*_max_=1.46 × 10^5^, corresponding to *g*=2*π* × 2.5 kHz.

To quantify the strength of the optomechanical coupling, in Fig. 3 we measure the cooperativity *C* between the mechanical resonator and the cavity. Together with initial phonon occupancy of the mechanical resonator 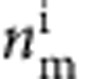, several criteria can be conveniently expressed with *C*, such that for reaching the quantum ground state of motion, 
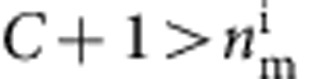
, or for reaching the radiation pressure shot noise limit, 
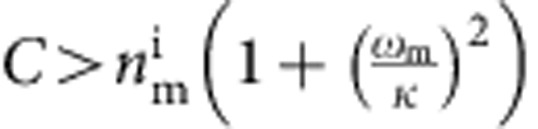
. To measure *C*, we use optomechanically induced transparency (OMIT)^10^,^28^,^29^, which allows one to directly determine the cooperativity with no free fit parameters. In OMIT, illustrated in Fig. 3a, the cavity is driven by a strong drive tone (*ω*_d_) while a second weak probe (*ω*_p_) is used to measure the cavity response. When driven on the red sideband (*ω*_d_=*ω*_0_−*ω*_m_), a transparency window appears within the broad resonance dip of the cavity reflection coefficient^10^. In the limit 
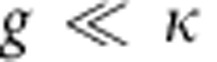
, the linewidth of the transparency window in 
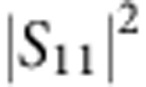
 is given by *γ*_m_(*C*+1) and the peak value by *C*/(*C*+1). Figure 3b shows an example of an OMIT measurement with drive-photon number *N*_d_=1.0 × 10^8^: from the broadened linewidth of the feature together with its near unity transmission, we extract *C*=94,500. Figure 3c shows the extracted *C* for different drive powers: at the maximum power sustained by the cavity, we achieve *C*_max_=1.46 × 10^5^.

### Microkelvin cooling of the membrane resonator

**Figure 4:**
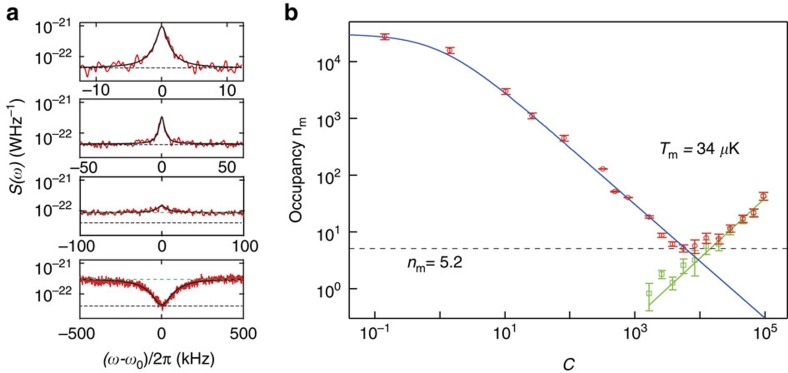
Microkelvin cooling of a millimetre-sized mechanical resonator. (**a**) Measured microwave PSD *S*(*ω*) with red-sideband driving (top to bottom: *C*=324, 785, 3,810 and 94,500). Black dashed line: noise floor *n*_add_ of the amplifier chain. As the cooperativity *C* of the cooling tone is increased, *S*(*ω*_0_) is reduced and the mechanical linewdith is broadened. Initially, the thermal noise of the membrane appears as a peak with decreasing height. At higher *C* (lower two panels), the noise floor outside of the mechanical linewidth (green dashed line) begins to increase above *n*_add_, indicating the presence of cavity noise. In the lowest panel, the output noise of the cavity is suppressed at *ω*_0_ due to correlations of the mechanical and cavity fluctuations. From the thermal peak and the cavity noise background level (green dashed line), we extract both *n*_m_ and *n*_c_ for all cooling powers (**b**). As a function of *C*, the mechanical occupancy *n*_m_ (red circle) drops, closely following 
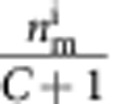
 (blue line) until the onset of cavity noise *n*_c_ (green squares) limits the final occupancy to a minimum of *n*_m_=5.2, corresponding to *T*_m_=34 μK. The error bars indicate the uncertainty of the data points and are calculated with the errors from the Lorentzian fit. The green line in **b** shows the expected *n*_c_ from the measured carrier sideband noise of the microwave generator.

The large cooperativity of our optomechanical set-up is, in principle, capable of cooling deep into the quantum ground state of motion if the mode is thermalized to the temperature of the fridge 
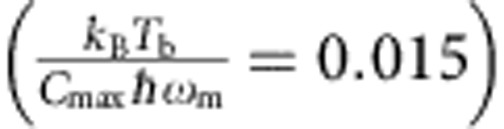
. To demonstrate the cooling of the resonator, in Fig. 4 we use the spectral density of the thermomechanical sideband to directly observe the phonon occupation of the membrane. While driving the cavity on the red sideband, the output microwave PSD *S*(*ω*_0_) at the cavity resonance frequency *ω*_0_ is given by:


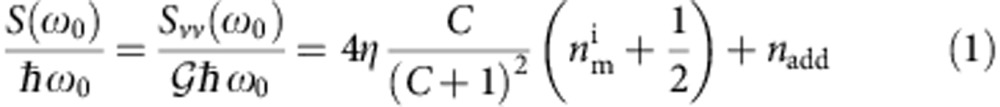


where *S*_*vv*_(*ω*_0_) is the measured microwave PSD on the spectrum analyser, 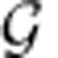 is the net gain of the signal path from the output of the cavity to the input of the spectrum analyser and *n*_add_ is the added photon noise quanta from the amplification chain referenced to the output of the cavity. By varying the bath temperature *T*_b_ and measuring *S*_*vv*_(*ω*), we obtain an absolute calibration of 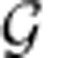, *n*_add_ and 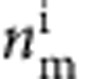 (details provided in Supplementary Fig. 3 and Supplementary Notes 3 and 4). At the base temperature of the refrigerator, we find that the membrane is thermalized to 
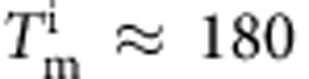
 mK, corresponding to 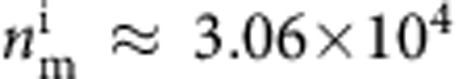. From the thermal calibration, we also extract *g*_0_=2*π* × 0.22 Hz, in good agreement with simulations (Supplementary Fig. 4 and Supplementary Note 5).

Figure 4a shows *S*(*ω*) for different cooperativities *C* of the cooling tone. As *C* increases, *S*(*ω*_0_) from the thermomechanical noise peak decreases, indicating that the mode temperature of the mechanical resonator is reduced. At larger cooling powers, however, although *S*(*ω*_0_) continues to drop, the noise floor of *S*(*ω*) outside the mechanical bandwidth begins to increase, and the peak in the PSD becomes a dip (bottom two panels of Fig. 4a). The increase in the noise floor is an indication of noise fluctuations of the cavity field^7^. Owing to correlations between the fluctuations of the cavity and of the mechanical resonator, the spectrum shows a suppression of the total PSD at the cavity frequency^30^.

To extract the mechanical occupation factor in the presence of cavity noise, it is no longer sufficient to look only at *S*(*ω*_0_). In particular, with sufficiently large *C* (and in the absence of other sources of heating that would increase 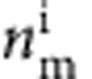 such as losses in the superconducting film or in the dielectric membrane or substrate), *S*(*ω*_0_) drops to the amplifier noise floor independent of the amount of cavity noise (equation (1)). This does not, however, imply that the mechanical mode is at zero temperature: due to the hybridization of the mechanical and optical fields, the final mechanical mode occupation in the presence of cavity noise is given by (Supplementary Note 6):


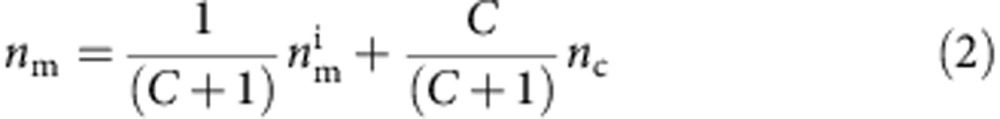


where *n*_c_ is the cavity noise measured in energy quanta. To find the final occupation *n*_m_, one must also determine *n*_c_. In the limit 
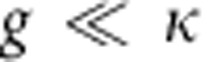
, cavity noise appears as an increase in the noise floor outside of the mechanical sideband (green dashed line in Fig. 4a) with a spectral density (see Supplementary Note 6 for the more general equation of *S*(*ω*)):


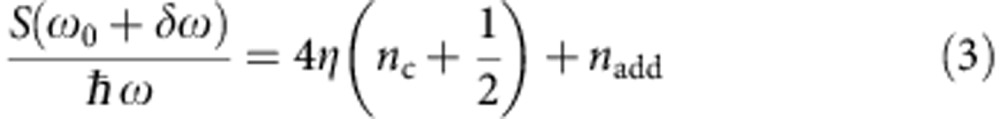


where 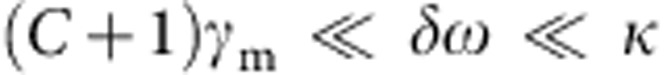. From this expression, one can extract the cavity occupation *n*_c_ and consequently *n*_m_ for all powers, shown in Fig. 4b. As *C* is increased, we find that *n*_m_ drops initially to a value of 5.2±0.7, corresponding to a mode temperature of 34±5 μK, beyond which the mechanical occupation begins to increase sharply due to heating from cavity noise. The temperature reported here is roughly a factor of two lower than that in recent experiments in the optical domain with silicon nitride membranes^23^,^24^ due to the very low frequency of our membrane.

Although the large cooperativity in our experiment should allow us to cool the membrane to *n*_m_=0.2 given the initial thermal occupation, in practice we are limited to *n*_m_=5.2±0.7 by cavity noise. To cool to lower occupation in future experiments, it is important to identify the source of this cavity noise. The green line in Fig. 4b shows the expected *n*_c_ due to the carrier sideband noise of our microwave signal generator (Supplementary Note 7). The good agreement with the observed cavity noise data suggests that our final occupation is limited by the spectral purity of the microwave tone used for the sideband cooling.

## Discussion

Having demonstrated the lowest temperature *T*_m_=34 μK reported to date for a mechanical resonator, we analyse the potential of this implementation to reach the deep quantum coherent coupling limit. In the current experiment, the cooling is limited by the classical sideband noise of the signal generator. Removing this noise with a tunable superconducting cavity with a linewidth of 10 kHz would already provide sufficient suppression to cool to a final occupation of 0.2. A second approach would be to increase *g*_0_ by shrinking the capacitor gap: reducing the membrane-antenna gap from 3 μm to 100 nm would increase *g*_0_ by a factor of 10^3^ and *C*_max_ by 10^6^. A third approach is to improve the cavity linewidth^27^, also yielding higher cooperativity at lower photon numbers. Finally, combining a smaller gap (*g*_0_∼300 Hz) with a better cavity (*κ*∼10 Hz) could yield ultrahigh cooperativities *C>*10^12^. Such a fully optimized design would also achieve single-photon strong coupling (*g*_0_>*κ*, *γ*_m_)^31^,^32^, enabling preparation and detection of non-classical states of motion such as Fock states or Schrödinger cat states with optomechanics.

In conclusion, we have developed a novel optomechanical system coupling the motion of a millimetre-sized membrane to a 3D microwave cavity. Exploiting the high coherence of the membrane and of the cavity, we achieve a cooperativity of *C*>1.4 × 10^5^ and perform sideband cooling of the millimetre-size membrane to 34 μK, corresponding to a thermal occupation *n*_m_=5.2. The scaling of this 3D optomechanical system offers the possibility to reach optomechanical couplings far beyond the state of the art, potentially entering the single-photon strong coupling regime in which a new generation of quantum experiments with mechanical objects would become possible.

## Methods

### Device preparation

We use commercial SiN_*x*_ membranes manufactured by Norcada. The membranes have the dimensions of 50 nm × 1 mm × 1 mm, and are supplied with a 5 × 5-mm Si frame. We deposit a 20-nm-thick film of Al on top of the membrane without covering the clamping edges by using a physical mask. On a separate sapphire substrate, we pattern two Al antenna pads with 80 nm of Al followed by the deposition of SiN_*x*_ spacer layer. To ensure the membrane does not come into contact with the substrate, a recess of 100 nm is etched. The metallized SiN_*x*_ membrane is then placed on top of the antenna pads to form a capacitor using a vacuum pick-and-place technique. A single drop of 0.1 μl of two-part epoxy is applied on the substrate to attach one corner of the membrane's Si frame to the substrate. Using the depth of focus to locate the vertical position of the membrane and of the bottom antenna pads while looking through the membrane with an optical microscope, we estimate the gap to be ∼3 μm. The gap is much larger than that designed in the spacer, most likely due to contamination from dust in the large contact area between the Si frame of the membrane and the substrate. The 3D microwave cavity is formed by closing two halves of a machined block made out of 6061-aluminium. The inner surface of the cavity is polished using a polishing paste, but is not chemically etched. The cavity is assembled by screwing the two halves of the cavity together with no sealing mechanism. The dimension of the whole cavity is 28 mm (*x*) × 28 mm (*y*) × 8 mm (*z*), with rounded corners of radius 1 mm in the *y*–*z* plane.

### Measurement

The bare frequency of the cavity without the antenna substrate and membrane is 7.4 GHz. The membrane attached to the antenna is placed at the centre of the cavity, coupling to the TE_110_ mode. We estimate the total mass of the membrane including the Al layer to be *m*=200 ng, corresponding to an estimated quantum zero-point fluctuation of *x*_zpf_=0.6 fm. Measurements are performed in a cryogen-free dilution refrigerator. To minimize cavity instability during the measurements, the sample is mounted on a mass-spring vibration isolation stage on the mixing chamber with resonance frequency of ∼1 Hz. During the cooling measurements, the pulse tube is temporarily turned off to minimize vibrations of the sample and the cables. Measurements are started 1.5 min after the switch-off and performed in an ∼2-min window before the temperature of the mixing chamber begins to increase.

## Additional information

**How to cite this article:** Yuan, M. *et al*. Large cooperativity and microkelvin cooling with a three-dimensional optomechanical cavity. *Nat. Commun*. 6:8491 doi: 10.1038/ncomms9491 (2015).

## Supplementary Material

Supplementary Information Supplementary Figures 1-4, Supplementary Notes 1-7 and Supplementary References
