# Supplementary material for: Large cooperativity and microkelvin cooling with a three-dimensional optomechanical cavity
Source: Nat Commun. 2015 Oct 9;6:8491. doi: 10.1038/ncomms9491 (PMC4633713; doi:10.1038/ncomms9491)
Supplement: Supplementary Information — Supplementary Figures 1-4, Supplementary Notes 1-7 and Supplementary References [file ncomms9491-s1.pdf]

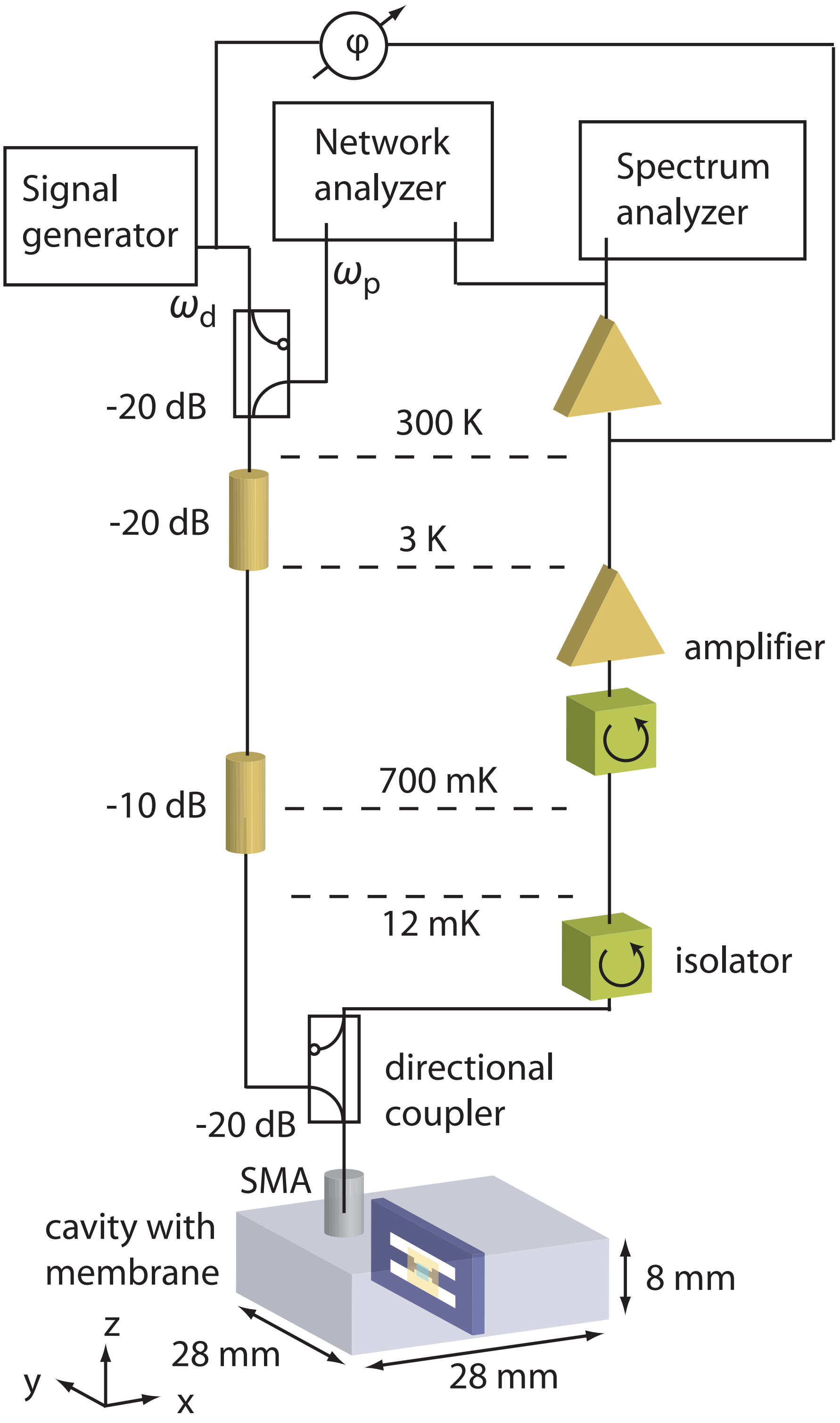

Supplementary Figure 1. Experimental setup for reflection measurements.

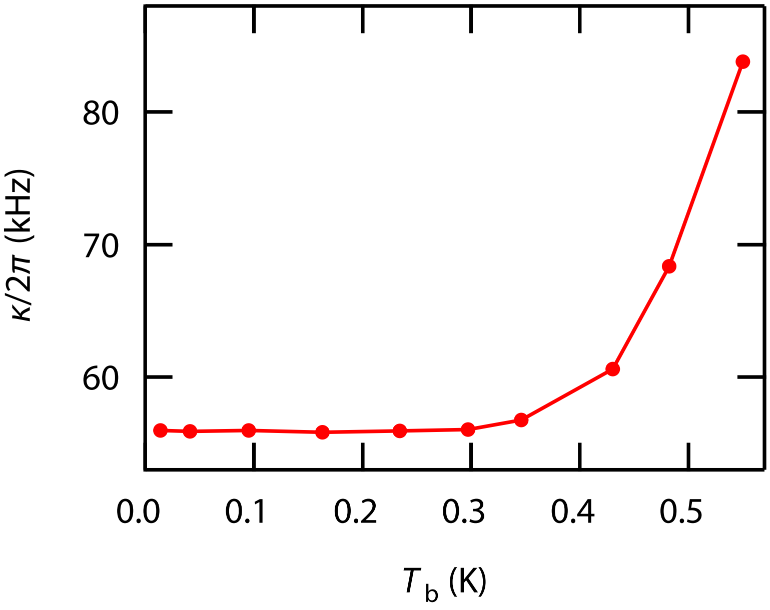

Supplementary Figure 2. Cavity decay rate κ as a function of cryostat base temperature $T_\mathbf{b}$. The line width of the cavity resonance starts to increase above 0.34 K.

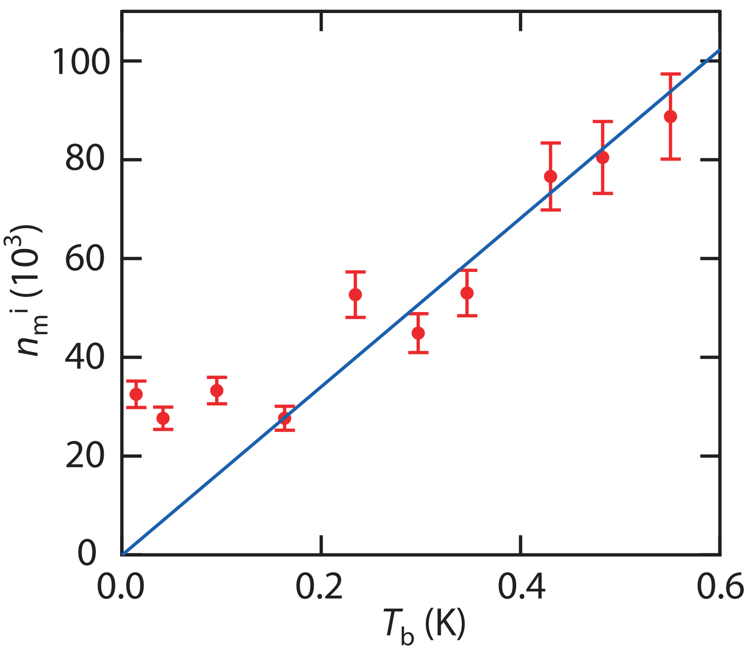

Supplementary Figure 3. Calibration of the initial thermal occupancy $n_{\mathbf{m}}^{\mathbf{i}}$ by varying the refrigerator temperature $T_{\mathbf{b}}$. The mode temperature of the membrane is thermalised at base temperature to $T_{\mathrm{m}}^{\mathrm{i}} \approx 180$ mK, corresponding to $n_{\mathrm{m}}^{\mathrm{i}} \approx 3.06 \times 10^4$. The error bars indicate the uncertainty of the data points, combining the fluctuations due to the temperature and the signal level.

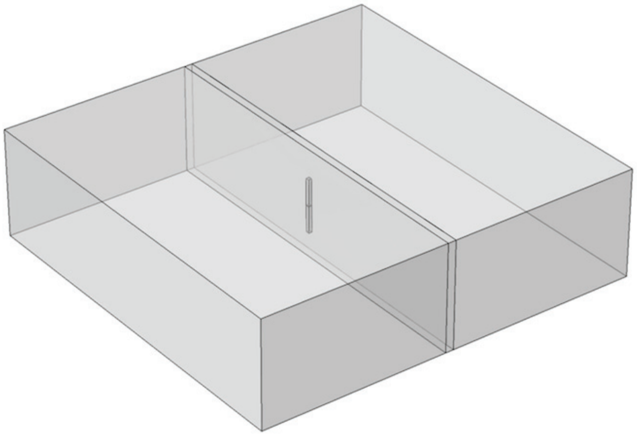

Supplementary Figure 4. COMSOL model showing a 3D cavity embedding an antenna.

Supplementary Note 1: Experimental setup

The sample is cooled down in a cryogen-free dilution refrigerator to a base temperature of 12 to 13 mK. Input microwave signals are attenuated at each thermal stage as illustrated by Supplementary Figure 1. At base temperature, the signal is injected into the cavity via the coupling port of a 20 dB directional coupler. The reflected power collected at the output port is amplified by a cold amplifier at the 3 K plate and subsequent amplifiers at room temperature and then detected by a network analyser or a spectrum analyser. Circulators are placed between the sample and the cold amplifier to isolate the sample from noise generated by the amplifier. A cancelling tone is applied using a directional coupler, a phase shifter and a continuous attenuator in order to reduce the amplitude of the strong drive tone before sending it to the room temperature amplifier. For the optomechanically-induced transparency (OMIT) experiment, a network analyser is used to generate the weak probe tone ω_{p} and a separate phase locked signal generator provides the strong drive tone ω_{d}.

Supplementary Note 2: Cavity characterisation

The cavity response $|S_{11}|$ in Fig. 2a in the main text is fitted with the following equation, taking into consideration the finite isolation of the directional coupler [1]:

$$|S_{11}(\omega)| = \left| \alpha e^{i\phi} + (1-\alpha)(1 - \frac{\kappa_{\mathrm{e}}}{i(\omega - \omega_0) + \frac{\kappa}{2}}) \right|, \tag{1}$$

where α is the isolation of the directional coupler.

Due to the low superconducting transition temperature of aluminium $T_c \sim 1.2$ K, the internal cavity quality factor is significantly reduced as the cryostat temperature T_{b} is raised above ∼0.34 K, and the line width κ is broadened, as shown in Supplementary Figure 2. This effect needs to be taken into account in the thermal calibration. As a function of temperature, we find that κ_{e} remains constant.

Supplementary Note 3: Calibration of the mode temperature

A thermal calibration is carried out to determine the initial (i.e. without cooling) phonon occupancy $n_{\mathrm{m}}^{\mathrm{i}} = \frac{1}{\exp \frac{\hbar\omega_{\mathrm{m}}}{k_{\mathrm{B}} T_{\mathrm{m}}^{\mathrm{i}}} - 1}$ of the mechanical resonator, k_{B} being the Boltzmann constant and $T_{\mathrm{m}}^{\mathrm{i}}$ the initial mode temperature. We send in a carrier signal at ω_0 to avoid any back-

action effects and measure the total power of its mechanical sideband at different cryostat temperature T_b. The total power generated by the thermal motion of the membrane is then used to extract the mechanical mode temperature T_m^i. We use a resolution bandwidth (RBW) of 1 Hz. In the limit of RBW$\gg \gamma_\mathrm{m}$, the total thermal power can be directly read out from the peak height. For $T_\mathrm{b} > 0.34$ K, ω_0 and κ changes significantly due to the low critical temperature of Al, which has to be taken into account. We plot the converted n_m^i as a function of refrigerator temperature T_b in Supplementary Figure 3. From the fitting line that passes the original point, at the base temperature the membrane is thermalised to $T_\mathrm{m}^\mathrm{i} \approx 180$ mK and $n_\mathrm{m}^\mathrm{i} \approx 3.06 \times 10^4$. The mode temperature is significantly higher than the base temperature of the refrigerator, which is probably an inevitable challenge one is faced with when working with a low-frequency (< 1 MHz) mechanical resonator.

Supplementary Note 4: Power calibration

The photon number in the cavity corresponding to the input power at the cavity P_in at frequency ω is calculated with

$$N = \frac{P_\mathrm{in}}{\hbar\omega} \cdot \frac{\kappa_\mathrm{e}}{(\frac{\kappa}{2})^2 + (\omega - \omega_0)^2}. \tag{2}$$

While driving the cavity on resonance, the sideband power due to the thermal motion is expressed as

$$\begin{aligned} P_\mathrm{side} =& P_\mathrm{in} \left(\frac{g_0}{x_\mathrm{zpf}}\right)^2 \cdot \frac{k_\mathrm{B}T}{m\omega_\mathrm{m}^2} \cdot \frac{(\frac{\kappa_\mathrm{e}}{2})^2}{(\frac{\kappa}{2})^2 + (\omega - \omega_\mathrm{r,b})^2} \frac{1}{(\frac{\kappa}{2})^2 + (\omega - \omega_0)^2} \\ =& P_\mathrm{in} \cdot g_0^2 \cdot 2n_\mathrm{m} \cdot \frac{(\frac{\kappa_\mathrm{e}}{2})^2}{(\frac{\kappa}{2})^2 + (\omega - \omega_\mathrm{r,b})^2} \frac{1}{(\frac{\kappa}{2})^2 + (\omega - \omega_0)^2}, \end{aligned} \tag{3}$$

where g_0 is again the single-photon coupling rate, k_B the Boltzmann constant and n_m the mechanical occupancy. The sideband frequency of interest is either ω_r or ω_b.

The loss of the input line $\mathcal{L}$ and the gain of the output line $\mathcal{G}$ are calibrated as the following: while driving the cavity on resonance ($\omega = \omega_0$) with power P'_in from the signal generator, the thermomechanical power measured by the spectrum analyser at the mechanical sidebands can be written as:

$$\begin{aligned} P_\mathrm{side} =& P'_\mathrm{in} \cdot g_0^2 \cdot 2n_\mathrm{m} \left(\frac{\kappa_\mathrm{e}}{\kappa}\right)^2 \frac{1}{(\frac{\kappa}{2})^2 + \omega_\mathrm{m}^2} \cdot \mathcal{L} \cdot \mathcal{G} \\ =& P'_\mathrm{in} \cdot g_0^2 \cdot \beta. \end{aligned} \tag{4}$$

The combined value of $\mathcal{L} \cdot \mathcal{G}$ is determined by the network analyser. Thermal occupation can be measured by sweeping the bath temperature as described in the previous section. All the parameters being known in β, single-photon coupling strength g_0 can be extracted. This is equivalent to the single-photon coupling strength calibration the frequency modulation (FM) technique described in [8], where the FM peak of the carrier wave provides a side-by-side reference of $\mathcal{L} \cdot \mathcal{G}$. Using this approach, we measure the single-photon coupling strength to be $g_0 = 0.22$ Hz. This further allows us to calibrate the corresponding photon number N by using the experimentally obtained photon-enhanced $g = g_0\sqrt{N}$ (from cooperativity measurements). Subsequently, by rewriting Supplementary Equation 2 as

$$N = \frac{P'_{\text{in}}}{\hbar\omega} \cdot \frac{\kappa_{\text{e}}}{(\frac{\kappa}{2})^2 + (\omega - \omega_0)^2} \cdot \mathcal{L}, \tag{5}$$

we calibrate the total input attenuation $\mathcal{L} = 70$ dB and correspondingly the output gain $\mathcal{G} = 73.5$ dB. The added noise of the output chain is calibrated to be $n_{\text{add}} = 12$ corresponding a noise temperature of approximately 2.6 K.

Supplementary Note 5: COMSOL RF simulations to estimate g_0

To estimate the single-photon coupling rate g_0 of the 3D-cavity-membrane system, we model the problem with a "variable capacitor in cavity" structure and numerically calculate the cavity frequency ω_0 with COMSOL. The following relation is used to derive g_0:

$$g_0 = \frac{\delta\omega_0}{\delta C_1} \cdot \delta C_2 \tag{6}$$

where δC_1 is the change in capacitance by varying the gap between the two antenna rods, $\delta\omega_0$ the subsequent frequency shift and δC_2 the change in capacitance due to zero-point fluctuation x_{zpf} of the membrane. δC_2 can be expressed as $\delta C_2 = \frac{\epsilon_0 A_2}{d^2} x_{\text{zpf}}$, where ϵ_0 is the free space permittivity, A_2 the area of the membrane and d the gap between the membrane and the antenna.

The geometry of the COMSOL model is shown in Supplementary Figure 4. The dimension of the 3D cavity is 28 mm×28 mm×8 mm. There is a 0.6 mm thick sapphire substrate at the centre of the cavity. The antenna is simplified to two Al rods with a cross section of 0.25 mm×0.25 mm, the length of the antenna being 4 mm and their gap is varied between 20 and 30 μm, changing the parallel-plate capacitance between them. Although this geometry is different from the actual geometry of the antenna, it results in comparable

frequency pull of ω_0, indicating that the capacitance participation ratio in the equivalent lumped-element circuit is similar. For a membrane of 1 mm×1 mm, a gap of $d = 3$ μm and $x_{\rm zpf} = 0.6$ fm the resulting coupling strength is $g_0 \approx 0.36$ Hz. Since g_0 is inversely proportional to the square of d, if d can be reduced to 30 nm, g_0 can be increased to 3.6 kHz.

Supplementary Note 6: Quantum noise of optomechanical cooling

The linearised Hamiltonian of the optomechanical system in a frame rotating at the drive tone frequency $\omega_{\rm d}$ can be written as [2–6]:

$$\hat{H} = -\hbar\Delta\hat{a}^\dagger\hat{a} + \hbar\omega_{\rm m}\hat{b}^\dagger\hat{b} - \hbar g(\hat{a}^\dagger + \hat{a})(\hat{b}^\dagger + \hat{b}), \tag{7}$$

where $\Delta = \omega_{\rm d} - \omega_0$, $\hat{a}^\dagger(\hat{a})$ the creation (annihilation) operator for the cavity field variation and $\hat{b}^\dagger(\hat{b})$ the creation (annihilation) operator for the mechanical mode. The optomechanical coupling $g = g_0\sqrt{N}$ is enhanced by the number of photons N, g_0 being the single-photon coupling rate.

We obtain the Heisenberg-Langevin equations

$$\begin{aligned}
\dot{\hat{a}}(t) &= \left(i\Delta - \frac{\kappa}{2}\right)\hat{a}(t) + ig(\hat{b}(t) + \hat{b}^\dagger(t)) + \sum_{j=\mathrm{e},0}\sqrt{\kappa_j}\hat{\xi}_j(t) \\
\dot{\hat{b}}(t) &= \left(-i\omega_{\rm m} - \frac{\gamma_{\rm m}}{2}\right)\hat{b}(t) + ig(\hat{a}(t) + \hat{a}^\dagger(t)) + \sqrt{\gamma_{\rm m}}\hat{\xi}_{\rm m}(t)
\end{aligned}$$

and their Hermitian conjugates

$$\begin{aligned}
\dot{\hat{a}}^\dagger(t) &= \left(-i\Delta - \frac{\kappa}{2}\right)\hat{a}^\dagger(t) - ig(\hat{b}(t) + \hat{b}^\dagger(t)) + \sum_{j=\mathrm{e},0}\sqrt{\kappa_j}\hat{\xi}_j(t) \\
\dot{\hat{b}}^\dagger(t) &= \left(i\omega_{\rm m} - \frac{\gamma_{\rm m}}{2}\right)\hat{b}^\dagger(t) - ig(\hat{a}(t) + \hat{a}^\dagger(t)) + \sqrt{\gamma_{\rm m}}\hat{\xi}_{\rm m}(t),
\end{aligned}$$

where κ and $\gamma_{\rm m}$ are the decay rates of the cavity and the mechanical resonator respectively. The noise operators satisfy the following relationships: $\langle\hat{\xi}^\dagger_{\rm m}(t)\hat{\xi}_{\rm m}(0)\rangle = n^{\rm i}_{\rm m}\delta(t)$, $\langle\hat{\xi}_{\rm m}(t)\hat{\xi}^\dagger_{\rm m}(0)\rangle = (n^{\rm i}_{\rm m} + 1)\delta(t)$; $\langle\hat{\xi}^\dagger_{\rm e}(t)\hat{\xi}_{\rm e}(0)\rangle = n_{\rm e}\delta(t)$, $\langle\hat{\xi}_{\rm e}(t)\hat{\xi}^\dagger_{\rm e}(0)\rangle = (n_{\rm e} + 1)\delta(t)$; $\langle\hat{\xi}^\dagger_0(t)\hat{\xi}_0(0)\rangle = n_0\delta(t)$, $\langle\hat{\xi}_0(t)\hat{\xi}^\dagger_0(0)\rangle = (n_0 + 1)\delta(t)$; $n_{\rm e}\kappa_{\rm e} + n_0\kappa_0 = n_{\rm c}\kappa$.

We will rewrite this in matrix form, setting

$$v(t) := \begin{pmatrix} \hat{a}(t) \\ \hat{a}^\dagger(t) \\ \hat{b}(t) \\ \hat{b}^\dagger(t) \end{pmatrix} \qquad w(t) := \begin{pmatrix} \sum_{j=\mathrm{e},0} \sqrt{\kappa_j}\hat{\xi}_j(t) \\ \sum_{j=\mathrm{e},0} \sqrt{\kappa_j}\hat{\xi}_j(t) \\ \sqrt{\gamma_\mathrm{m}}\hat{\xi}_\mathrm{m}(t) \\ \sqrt{\gamma_\mathrm{m}}\hat{\xi}_\mathrm{m}(t) \end{pmatrix}$$

and

$$A := \begin{pmatrix} i\Delta - \frac{\kappa}{2} & 0 & ig & ig \\ 0 & -i\Delta - \frac{\kappa}{2} & -ig & -ig \\ ig & ig & -i\omega_\mathrm{m} - \frac{\gamma_\mathrm{m}}{2} & 0 \\ -ig & -ig & 0 & i\omega_\mathrm{m} - \frac{\gamma_\mathrm{m}}{2} \end{pmatrix}$$

thus we get

$$v'(t) = Av(t) + w(t).$$

Using Fourier transforms $\mathcal{F}(f(t)) = f(\omega) \equiv \int_{-\infty}^{\infty} f(t)e^{i\omega t}\,\mathrm{d}t$ we have

$$-i\omega v(\omega) = Av(\omega) + w(\omega)$$

which has the solution

$$v(\omega) = (-i\omega I - A)^{-1}\, w(\omega) = B^{-1}w(\omega),$$

$$B = \begin{pmatrix} -i\omega - i\Delta + \frac{\kappa}{2} & 0 & -ig & -ig \\ 0 & -i\omega + i\Delta + \frac{\kappa}{2} & ig & ig \\ -ig & -ig & -i\omega + i\omega_\mathrm{m} + \frac{\gamma_\mathrm{m}}{2} & 0 \\ ig & ig & 0 & -i\omega - i\omega_\mathrm{m} + \frac{\gamma_\mathrm{m}}{2} \end{pmatrix}$$

$$\equiv \begin{pmatrix} 1/\chi_c & 0 & -ig & -ig \\ 0 & 1/\bar{\chi}_c & ig & ig \\ -ig & -ig & 1/\chi_\mathrm{m} & 0 \\ ig & ig & 0 & 1/\bar{\chi}_\mathrm{m} \end{pmatrix}.$$

For cooling we use $\Delta = -\Omega_{\mathrm{m}}$ and let $\delta = \omega - \Omega_{\mathrm{m}}$. Applying the rotating wave approximation, omitting contribution from $\bar{\chi}_c$, $\bar{\chi}_{\mathrm{m}}$, we get

$$\hat{a}(\omega) = \frac{\chi_c \sum \sqrt{\kappa_i}\hat{\xi}_i + ig\chi_{\mathrm{m}}\chi_c\sqrt{\gamma_{\mathrm{m}}}\hat{\xi}_{\mathrm{m}}}{1 + g^2\chi_c\chi_{\mathrm{m}}} \tag{8}$$

$$\hat{b}(\omega) = \frac{\chi_{\mathrm{m}}\sqrt{\gamma_{\mathrm{m}}}\hat{\xi}_{\mathrm{m}} + ig\chi_{\mathrm{m}}\chi_c \sum \sqrt{\kappa_j}\hat{\xi}_j}{1 + g^2\chi_c\chi_{\mathrm{m}}}. \tag{9}$$

From the input-output theory the output field can be expressed as

$$\begin{aligned} \hat{a}_{\mathrm{out}} =& \hat{\xi}_e - \sqrt{\kappa_{\mathrm{e}}}\hat{a} \\ =& \left(1 - \frac{\chi_c\kappa_{\mathrm{e}}}{1 + g^2\chi_c\chi_{\mathrm{m}}}\right)\hat{\xi}_{\mathrm{e}} - \frac{\chi_c\sqrt{\kappa_{\mathrm{e}}\kappa_0}}{1 + g^2\chi_c\chi_{\mathrm{m}}}\hat{\xi}_0 - \frac{i\sqrt{\kappa_{\mathrm{e}}\gamma_{\mathrm{m}}}g\chi_c\chi_{\mathrm{m}}}{1 + g^2\chi_c\chi_{\mathrm{m}}}\hat{\xi}_{\mathrm{m}}. \end{aligned} \tag{10}$$

Since a cancelling tone is added, the detected field is modified as

$$\begin{aligned} \hat{a}_{\mathrm{out}} =& \hat{\xi}_e - \sqrt{\kappa_{\mathrm{e}}}\hat{a} - \hat{\xi}_e \\ =& \frac{\chi_c\kappa_{\mathrm{e}}}{1 + g^2\chi_c\chi_{\mathrm{m}}}\hat{\xi}_{\mathrm{e}} - \frac{\chi_c\sqrt{\kappa_{\mathrm{e}}\kappa_0}}{1 + g^2\chi_c\chi_{\mathrm{m}}}\hat{\xi}_0 - \frac{i\sqrt{\kappa_{\mathrm{e}}\gamma_{\mathrm{m}}}g\chi_c\chi_{\mathrm{m}}}{1 + g^2\chi_c\chi_{\mathrm{m}}}\hat{\xi}_{\mathrm{m}} \\ =& \sum_{k=\mathrm{e},0,\mathrm{m}} f_k(\omega)\hat{\xi}_k(\omega). \end{aligned} \tag{11}$$

The spectrum analyser detects the symmetric power spectral density (PSD) [7]

$$\begin{aligned} \frac{S(\omega)}{\hbar\omega} =& \frac{1}{2}\int_{-\infty}^{\infty} e^{i\omega t}\langle \hat{a}^\dagger_{\mathrm{out}}(0)\hat{a}_{\mathrm{out}}(t) + \hat{a}_{\mathrm{out}}(t)\hat{a}^\dagger_{\mathrm{out}}(0)\rangle \mathrm{d}t. \\ =& \frac{1}{2\pi}\frac{1}{2}\langle \hat{a}^\dagger_{\mathrm{out}}(-\omega)\hat{a}_{\mathrm{out}}(\omega) + \hat{a}_{\mathrm{out}}(\omega)\hat{a}^\dagger_{\mathrm{out}}(-\omega)\rangle. \end{aligned} \tag{12}$$

Strictly speaking this is for the lab frame, however in this case only the detunings enter the equations, therefore we could directly substitute in Supplementary Equation 8 and 11 and get

$$\frac{S(\omega)}{\hbar\omega} = \sum_{k=\mathrm{e},0,\mathrm{m}} |f_k(\omega)|^2 \left(n^{\mathrm{i}}_k + \frac{1}{2}\right), \tag{13}$$

$n^i_{\mathrm{e},0}$ being equivalent to $n_{\mathrm{e},0}$. Including the added noise from the amplification chain, we get

$$\frac{S(\omega)}{\hbar\omega} = \frac{g^2\kappa_{\mathrm{e}}\gamma_{\mathrm{m}}}{|g^2 + \left(\frac{\kappa}{2} - i\delta\right)\left(\frac{\gamma_{\mathrm{m}}}{2} - i\delta\right)|^2}\left(n^{\mathrm{i}}_{\mathrm{m}} + \frac{1}{2}\right) + \frac{|\frac{\gamma_{\mathrm{m}}}{2} - i\delta|^2\kappa_{\mathrm{e}}\kappa}{|g^2 + \left(\frac{\kappa}{2} - i\delta\right)\left(\frac{\gamma_{\mathrm{m}}}{2} - i\delta\right)|^2}\left(n_{\mathrm{c}} + \frac{1}{2}\right) + n_{\mathrm{add}}. \tag{14}$$

Note that the noise operators in the frequency domain satisfy the following relations: $\langle \hat{\xi}^\dagger_k(\omega)\hat{\xi}_k(\omega')\rangle = 2\pi n^{\mathrm{i}}_k\delta(\omega + \omega')$, $\langle \hat{\xi}_k(\omega)\hat{\xi}^\dagger_k(\omega')\rangle = 2\pi(n^{\mathrm{i}}_k + 1)\delta(\omega + \omega')$.

The final occupation can be found via equipartition [4, 5]:

$$\begin{aligned} 1+2n_{\mathrm{m}} =& \frac{\langle \hat{x}^2 \rangle}{x_{\mathrm{zpf}}^2} = \int_{-\infty}^{\infty} \frac{\mathrm{d}\omega}{2\pi} \frac{S_{xx}(\omega)}{x_{\mathrm{zpf}}^2} \\ =& \frac{1}{x_{\mathrm{zpf}}^2} \int_{-\infty}^{\infty} \frac{\mathrm{d}\omega}{2\pi} \int_{-\infty}^{\infty} \frac{1}{2} \langle \hat{x}(t)\hat{x}(0) + \hat{x}(0)\hat{x}(t) \rangle e^{i\omega t} \mathrm{d}t, \end{aligned} \tag{15}$$

where $\hat{x}(t) = x_{\mathrm{zpf}}(\hat{b}(t) + \hat{b}^{\dagger}(t))$, x_{zpf} being the zero-point fluctuation. Substituting in Supplementary Equation 9, using formulae of contour integrals and considering $\kappa^2 \gg 4g^2, \kappa\gamma_{\mathrm{m}}, \gamma_{\mathrm{m}}$, we find

$$n_{\mathrm{m}} \approx \frac{\kappa\gamma_{\mathrm{m}}}{4g^2 + \kappa\gamma_{\mathrm{m}}} n_{\mathrm{m}}^{\mathrm{i}} + \frac{4g^2}{4g^2 + \kappa\gamma_{\mathrm{m}}} n_{\mathrm{c}} = \frac{1}{C+1} n_{\mathrm{m}}^{\mathrm{i}} + \frac{C}{C+1} n_{\mathrm{c}}. \tag{16}$$

Supplementary Note 7: Noise from the signal generator

For the measurement shown in Fig. 4 of the main text, we use a Phase Matrix QuickSyn FSW-0020 microwave signal generator. We measure a sideband noise S_{ϕ} of -130 dBc/Hz at 120 kHz offset for a 5.1 GHz carrier signal. Cavity noise occupancy n_{c} contributed by the signal's own noise can be estimated by

$$n_{\mathrm{c}} = \frac{P_{\mathrm{in}} S_{\phi} \cdot \kappa_{\mathrm{e}}}{\hbar\omega} \frac{\kappa_{\mathrm{e}}}{(\kappa/2)^2}. \tag{17}$$

For a critically coupled cavity, we have

$$n_{\mathrm{c}} = \frac{P_{\mathrm{in}} \times 10^{-13}/\mathrm{Hz}}{\hbar\omega}. \tag{18}$$

The green line in Fig. 4c in the main text is plotted with the above equation.

Measurements were also performed using an a Agilent PSG-UNY low phase noise option microwave signal generator which was available to us for a short time. Although the phase noise of the PSG-UNY at an offset of 120 kHz is specified to be -137 dBc/Hz for 5 GHz carrier signals, the total sideband noise (amplitude and phase) was observed to be -132 dBc/Hz. Although we did not have time to perform a full thermal calibration of the setup with the PSG-UNY, we observed that the cavity noise n_{c} was about 2 dB lower with the PSG generator with no evidence of additional mode heating, implying that the final occupation with the PSG generator would be 4.3 phonons.

Supplementary References

[1] Singh, V. *et al.* Optomechanical coupling between a multilayer graphene mechanical resonator and a superconducting microwave cavity. *Nat. Nanotechnol.* **9**, 820–824 (2014).

[2] Aspelmeyer, M., Kippenberg, T. J. & Marquardt, F. Cavity optomechanics. *Rev. Mod. Phys.* **86**, 1391–1452 (2014).

[3] Marquardt, F., Chen, J., Clerk, A. & Girvin, S. Quantum theory of cavity-assisted sideband cooling of mechanical motion. *Phys. Rev. Lett.* **99**, 093902 (2007).

[4] Dobrindt, J. M., Wilson-Rae, I. & Kippenberg, T. J. Parametric normal-mode splitting in cavity optomechanics. *Phys. Rev. Lett.* **101**, 263602 (2008).

[5] Rocheleau, T. *et al.* Preparation and detection of a mechanical resonator near the ground state of motion. *Nature* **463**, 72–75 (2010).

[6] Teufel, J. D. *et al.* Sideband cooling of micromechanical motion to the quantum ground state. *Nature* **475**, 359–363 (2011).

[7] Clerk, A. A., Devoret, M. H., Girvin, S. M., Marquardt, F. & Schoelkopf, R. J. Introduction to quantum noise, measurement, and amplification. *Rev. Mod. Phys.* **82**, 1155–1208 (2010).

[8] Gorodetsky, M. L., Schliesser, A., Anetsberger, G., Deleglise, S. & Kippenberg, T. J. Determination of the vacuum optomechanical coupling rate using frequency noise calibration. *Opt. Express* **18**, 23236–23246 (2010).
